# Physiological response of *Kobresia pygmaea* to temperature changes on the Qinghai-Tibet Plateau

**DOI:** 10.1186/s12870-022-03428-9

**Published:** 2022-01-24

**Authors:** Haiyan Xu, Lisha Li, Nan Mao, Zipeng Gan, Shouye Xue, Xiaoming Li, Bo Zhang, Guimin Liu, Xiaodong Wu

**Affiliations:** 1 School of Environmental and Municipal Engineering, Lanzhou Jiaotong University, Lanzhou, China; 2 Cryosphere Research Station on the Qinghai-Tibet Plateau, State Key Laboratory of Cryospheric Science, Northwest Institute of Eco-Environment and Resources, Chinese Academy of Sciences, Lanzhou, China

**Keywords:** *Kobresia pygmaea*, Physiological characteristics, Seasonal changes, Osmotic adjustment substances, Antioxidant enzyme activity

## Abstract

**Background:**

The Qinghai-Tibetan Plateau is experiencing rapid climate warming, which may further affect plant growth. However, little is known about the plant physiological response to climate change.

**Results:**

Here, we select the *Kobresia pygmaea*, an important perennial Cyperaceae forage, to examine the physiological indices to temperature changes in different growing months. We determined the contents of malondialdehyde, proline, soluble sugars, superoxide dismutase, peroxidation, and catalase activity in leaves and roots of *Kobresia pygmaea* at 25℃, 10℃, 4℃ and 0℃ from June to September in 2020. The results showed that the content of osmotic adjustment substances in the leaves and roots of *Kobresia pygmaea* fluctuated greatly with experimental temperature in June and September. The superoxide dismutase activity in the leaves and roots of the four months changed significantly with temperatures. The peroxidation activity in the leaves was higher than that in the roots, while the catalase activity in leaves and roots fluctuates greatly during June, with a relative stable content in other months. Membership function analysis showed that higher temperatures were more harmful to plant leaves, and lower temperatures were more harmful to plant roots. The interaction of organs, growing season and stress temperature significantly affected the physiological indicators.

**Conclusions:**

The physiological indicators of *Kobresia pygmaea* can actively respond to temperature changes, and high temperature can reduce the stress resistance *Kobresia pygmaea*. Our findings suggest that the *Kobresia pygmaea* has high adaptability to climate warming in the future.

**Supplementary Information:**

The online version contains supplementary material available at 10.1186/s12870-022-03428-9.

## Background

According to IPCC, the land surface air temperature increased by1.41 °C (1.31–1.51 °C) during 1880–2018 [1], and the temperature rises in high latitudes and high-altitude areas were even greater [2, 3]. Climate change has important effects on both morphological structure and physiological characteristics of plants [4]. In fact, climate warming in recent years has caused changes in vegetation phenology [5].The increasing temperature advances flowering time of plants, and a 2.5 °C increase in temperature of will cause the advance 5-25 days for flowering time of different species [6, 7]. Climate warming can also change the physiological metabolism of plant cells. High temperature can damage plant biomembrane structure, decrease cell water potential, increase relative permeability of lipid membrane and electrolyte extravasation. These changes can lead to physiological, biochemical and metabolic disorders in plants [8]. Although the responses of plant phenology, grassland community biomass, species composition and diversity to climate change have been extensively studied, there are few reports to the impact of climate change on plant physiology [9–11]. To cope with temperature stress, plants have evolved a variety of efficient mechanisms that allow them to adapt to the adverse conditions [12, 13]. This adaptive process involves a number of biochemical and physiological changes, including increased levels of proline, soluble sugars, and malondialdehyde, as well as enzyme activities [14]. These indices have been widely used to investigate the plant physiology response to high temperature and low temperature on plant seedlings, as well as plant leaves [15].

The Qinghai-Tibet Plateau (QTP) is a unique eco-region because of its high elevation [16]. The QTP is mostly covered by land cover types of typical alpine meadow and steppe. These vegetation is not only providing important ecological functions such as carbon storage, water resource regulation, climate control at a global scale, but also providing critical ecosystem services such as pastoral production, cultural inheritance at local and regional scales [17, 18]. Therefore, it is important to understand the response mechanisms of vegetation to temperature change. Based on the remote sensing data, it was concluded that the vegetation cover showed an overall increasing trend since the 1980 s [19, 20], the QTP, a temperature-limited ecosystem, the vegetation growth is sensitive climate change [21]. It is reasonable to infer that the climate change not only affects vegetation coverage and biomass, and it may also have effects on the physiology of plant.

The grassland area of the QTP reaches 1.28 × 10^6^ km^2^, accounting for about 50% of the total area. The alpine meadows and alpine grasslands account 49.3% and 44.9% of the total grassland area [22]. For the meadows, the *Kobresia* are the dominant species, and 56% of the alpine meadows are mainly consisted of these species. The *Kobresia* are the main forage grasses on the QTP [23]. The physiological effects of climate change on the leaves have been investigated using experimental warming [24]. It has been found that the leaf length and number of *Kobresia pygmaea* increased with temperature, but the content of malondialdehyde, free proline and antioxidant enzyme activity showed no significant changes [25]. Although the *Kobresia pygmaea* showed physiological response to climate change in the leaves, there are no comparative studies on the physiology of different organs of *Kobresia pygmaea* in different growing seasons. These knowledge gap hinder our understanding of effects of climate change on vegetation in alpine cold regions.

In this study, using laboratory experiments, *Kobresia pygmaea* samples which were collected in different growing seasons were cultivated at 23 °C, 10 °C, 4 °C and 0 °C in 2020. The content of malondialdehyde, free proline and soluble sugars in the leaves and roots were determined. We also measured the related antioxidant enzyme activities. The main goal of this study is to explore the physiological response of *Kobresia pygmaea* to temperature changes. Specifically, there are two scientific questions: 1) What are the seasonal physiological changes of *Kobresia pygmaea* leaves and roots under natural conditions? 2) Under different temperatures, what are the differences of physiological changes among different growing season of *Kobresia pygmaea*? Since the physiological ability to adapt to the environment can help plants to cope with environmental changes [26], the results will be helpful to understand the physiological adaptability of plants in alpine regions and their response to climate change in the future.

## Results

### Changes in malondialdehyde (MDA)

**Fig. 1:**
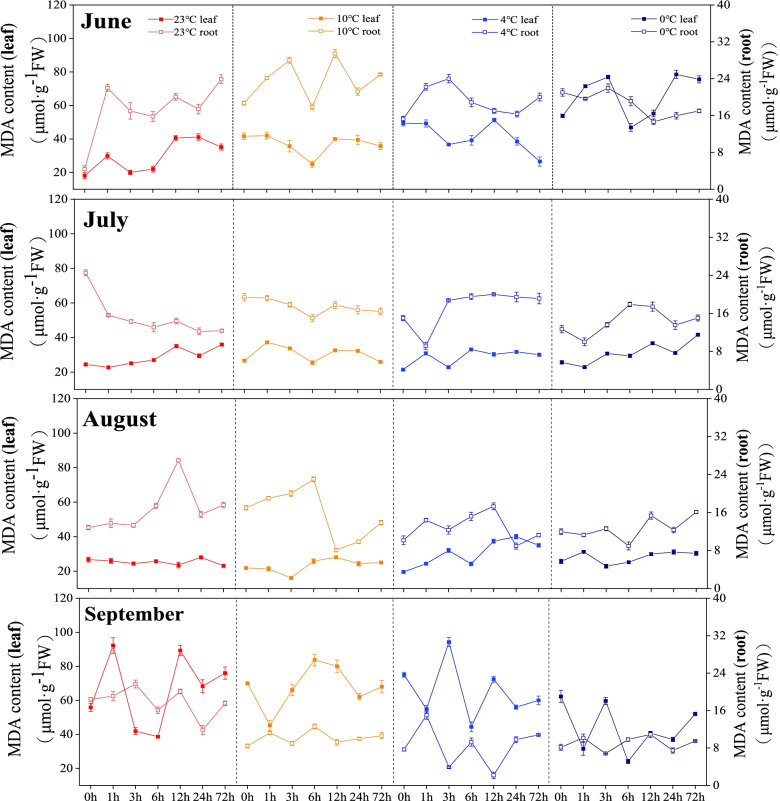
Changes in malondialdehyde content of *Kobresia pygmaea* under different temperatures. The x-axis represents the time period

The content of malondialdehyde in leaves of *Kobresia pygmaea* fluctuated greatly among the different months. In June, the MDA content both in leaves and roots increased slightly with the decreasing incubation temperature. In July and August, the MDA contents in roots showed slightly decreased with the decreasing incubation temperature, while the changing trend of the leaves were opposite. In September, the mean MDA contents in roots and leaves also showed decreasing trends from 23 °C to 0 °C (Fig. 1).

### Changes in osmotic regulation

**Fig. 2:**
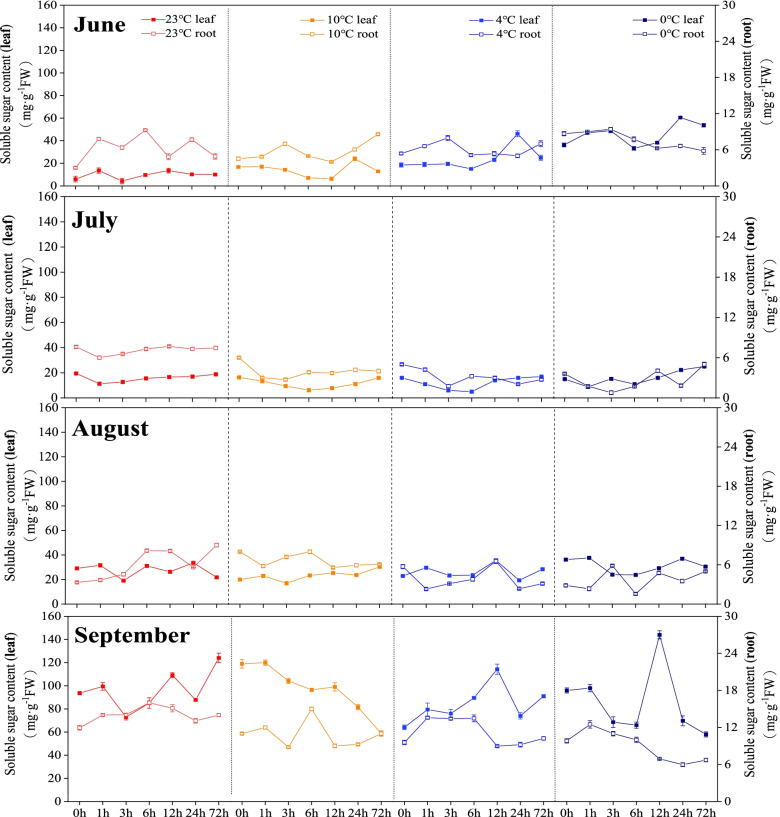
Changes in soluble sugar content of *Kobresia pygmaea* under different temperatures. The x-axis represents the time period

The soluble sugar contents both in leaves and roots in June showed similar values in June. In July, the soluble sugar contents showed the lowest values at 0℃. In August, the soluble sugar content in the leaves and roots were relatively stable at different temperatures. In September, the soluble sugar contents in leaves varied considerably, while the contents in roots were lower at 0℃ in comparison with other temperatures (Fig. 2).

**Fig. 3:**
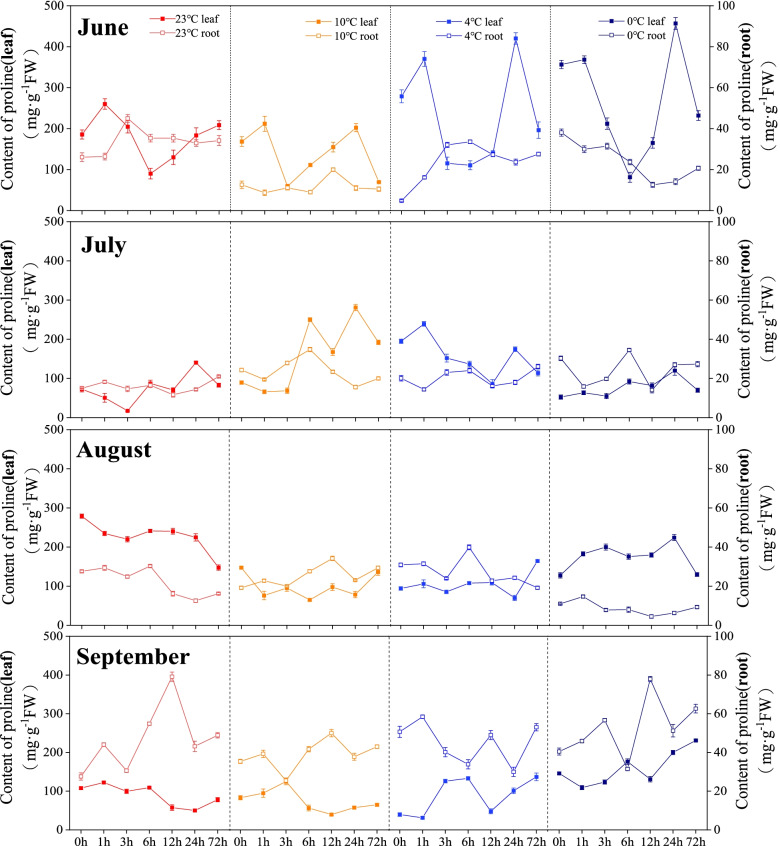
Changes in free proline content of *Kobresia pygmaea* under different temperatures. The x-axis represents the time period

In June, the free proline contents in roots and leaves showed relatively stable changes at 23℃and 10℃. In July, the free proline contents in leaves and roots were higher at 10 °C and 4 °C. In August, the free proline contents were higher at 23 ℃, and the lowest values in roots were recorded at 0 ℃. For the samples collected in September, the free proline contents showed great variations, and the free proline contents in leaves and roots gradually increased with the decreasing temperature (Fig. 3).

### Changes in enzyme activity

**Fig. 4:**
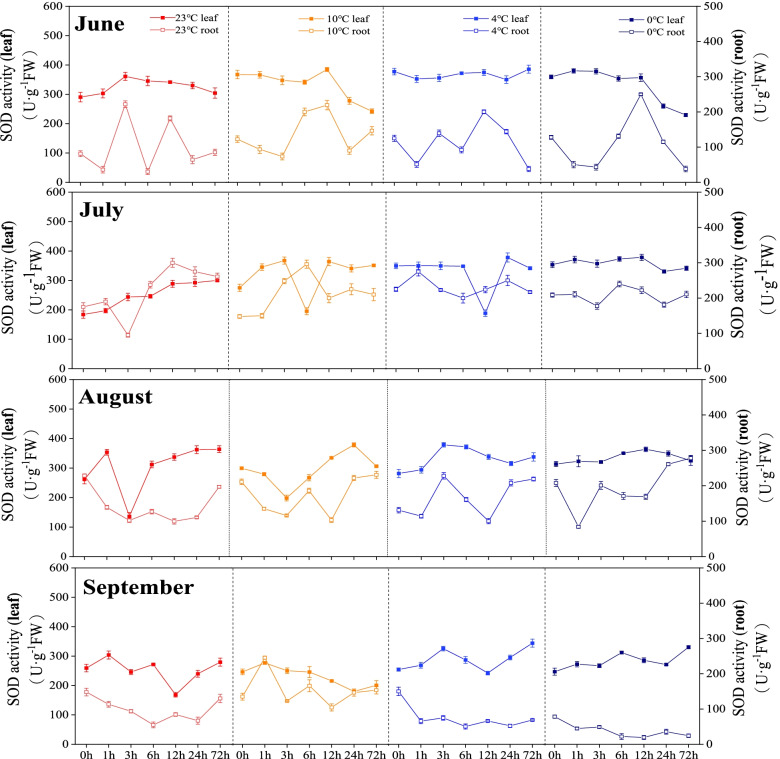
Changes in superoxide dismutase activity of *Kobresia pygmaea* under different temperatures. The x-axis represents the time period

In June, the Superoxide dismutase (SOD) activities in roots showed a greater fluctuation than those in leaves. In July and August, the SOD activities both in leaves and roots showed no significant changes at different temperatures. In September, the SOD activities in roots were largely lower at 4 °C and 0 °C than those at 23 °C and 10 °C, while the SOD activities contents in leaves increased slightly with the decreasing incubation temperature (Fig. 4).

**Fig. 5:**
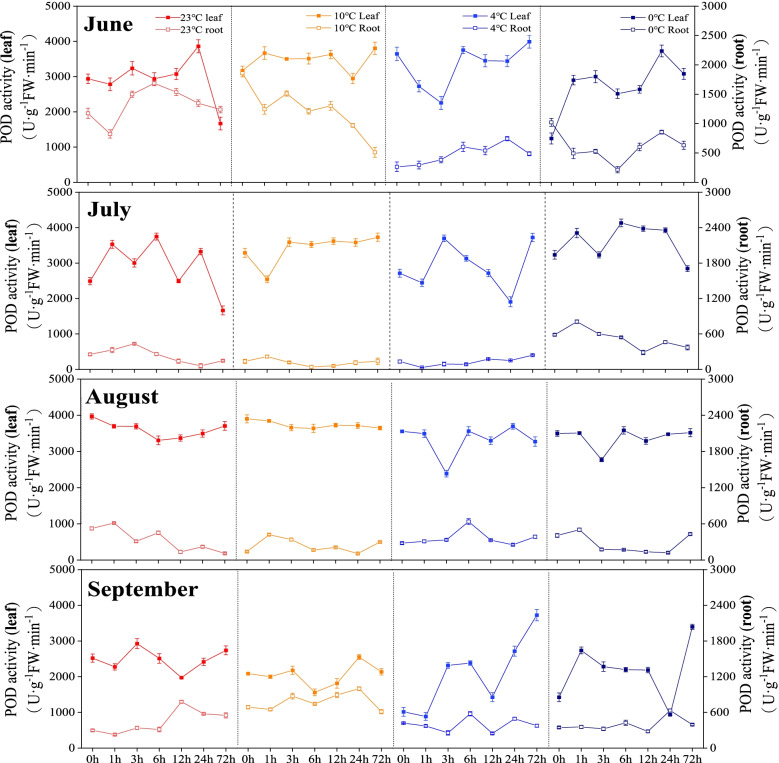
Changes in peroxidase activity of *Kobresia pygmaea* under different temperatures. The x-axis represents the time period

**Fig. 6:**
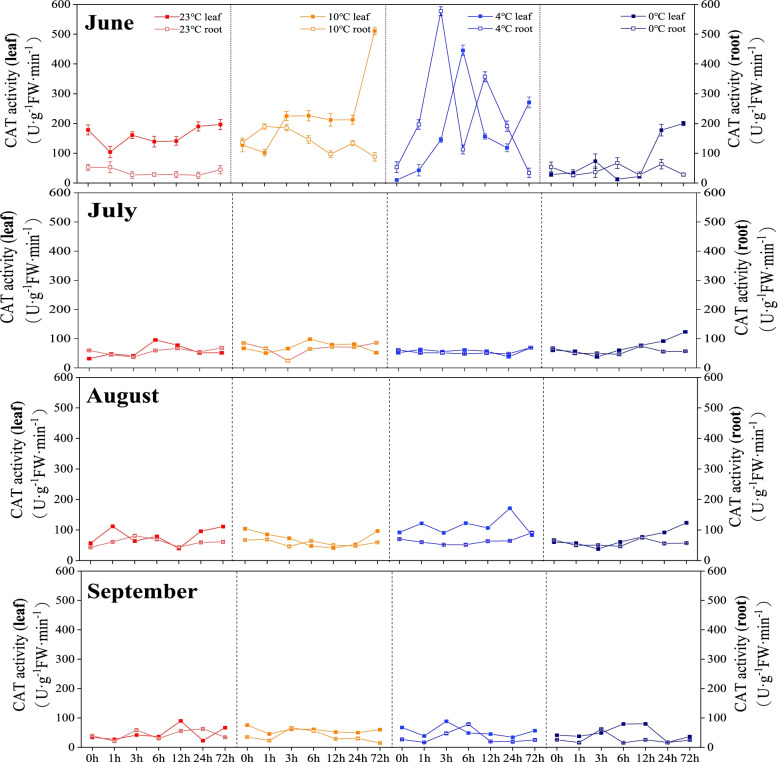
Changes in Catalase activity of *Kobresia pygmaea* under different temperatures. The x-axis represents the time period

The peroxidase (POD) activities in the roots in June were the lowest at 4 ℃ and 0 ℃. In July and August, the POD activities in leaves were significantly higher than those in roots. In September, the POD activities fluctuated considerably at all the four temperatures (Fig. 5). The Catalyse (CAT) activities in leaves were higher in June than other three months. From July to September, the CAT showed only slightly changes at different temperatures (Fig. 6).

### Membership function of plant organs in different growing seasons at different temperatures

The membership function values were shown in Table 1. The greater membership function values, the higher osmotic adjustment substances accumulate, the higher the antioxidant enzyme activity, and the smaller the membrane damage in *Kobresia pygmaea*. According to the average values, the most harmful temperature to the leaves of plants is 23℃ in June and July. The most harmful temperature to the leaves is 10℃ in August and September (Table 1). In June and July, the most harmful temperature to the roots is 4℃. In August and September, the most harmful temperature to the roots is 0℃ (Table 2).

**Table 1:** Membership function values of physiological indices in *Kobresia pygmaea* leaves at different temperatures

Leaf	MDA	Pro	SS	SOD	POD	CAT	Average
June 23℃	0.19	0.31	0.09	0.61	0.61	0.30	**0.35**
10℃	0.31	0.20	0.17	0.66	0.81	0.44	0.43
4℃	0.39	0.44	0.35	0.88	0.76	0.32	0.52
0℃	0.78	0.53	0.73	0.65	0.54	0.08	0.55
July 23℃	0.35	0.22	0.55	0.34	0.50	0.27	**0.37**
10℃	0.45	0.54	0.32	0.70	0.71	0.43	0.52
4℃	0.36	0.53	0.36	0.75	0.50	0.27	0.46
0℃	0.48	0.27	0.65	0.89	0.78	0.44	0.59
August 23℃	0.39	0.76	0.51	0.69	0.77	0.31	0.57
10℃	0.30	0.16	0.30	0.65	0.85	0.24	**0.42**
4℃	0.60	0.19	0.44	0.80	0.59	0.55	0.53
0℃	0.50	0.51	0.69	0.82	0.63	0.61	0.63
September 23℃	0.60	0.29	0.44	0.48	0.56	0.39	0.46
10℃	0.62	0.22	0.45	0.34	0.41	0.56	**0.43**
4℃	0.59	0.28	0.30	0.68	0.42	0.51	0.46
0℃	0.28	0.64	0.32	0.66	0.45	0.43	0.46

Note: *SS *Soluble sugar content

**Table 2:** Membership function values of physiological indices in *Kobresia pygmaea* roots at different temperatures

Root	MDA	Pro	SS	SOD	POD	CAT	Average
June 23℃	0.51	0.72	0.51	0.32	0.68	0.02	0.46
10℃	0.76	0.18	0.42	0.48	0.62	0.21	0.44
4℃	0.59	0.47	0.48	0.38	0.16	0.35	**0.40**
0℃	0.57	0.49	0.83	0.35	0.25	0.03	0.42
July 23℃	0.39	0.19	0.92	0.61	0.26	0.51	0.48
10℃	0.54	0.52	0.45	0.57	0.11	0.69	0.48
4℃	0.53	0.38	0.34	0.66	0.13	0.49	**0.42**
0℃	0.33	0.55	0.27	0.55	0.63	0.65	0.50
August 23℃	0.46	0.51	0.61	0.31	0.44	0.45	0.46
10℃	0.41	0.58	0.69	0.45	0.25	0.41	0.47
4℃	0.25	0.65	0.31	0.42	0.48	0.53	0.44
0℃	0.24	0.12	0.29	0.58	0.32	0.32	**0.31**
September 23℃	0.80	0.40	0.80	0.35	0.28	0.45	0.51
10℃	0.41	0.25	0.49	0.59	0.72	0.34	0.47
4℃	0.32	0.36	0.52	0.25	0.21	0.29	0.33
0℃	0.35	0.50	0.30	0.09	0.22	0.19	**0.27**

Note: *SS *Soluble sugar content

## Multi-factor analysis of variance of physiological indices

Using multi-factor analysis of variance, the effects of organ, month, and temperature factors on physiological indices were analyzed (Table 3). Obviously, the organ has significant effects on all the indices (*p*<0.01), and the month has effects on most of the indices (*p*<0.01). The cultivate temperature has significant effects on Catalyze activity. The interaction of organ and cultivate temperature, month and cultivate temperature has significant effects on SOD and Catalyze activities.

**Table 3:** * F* values of Multi-factor analysis of variance of physiological indicators of *Kobresia pygmaea*

Factor	MDA	Pro	SS	SOD	POD	CAT
Organ	**184.655**^b^	**210.907**^b^	**99.277**^b^	**331.031**^b^	**969.739**^b^	**7.532**^b^
Growing season	**9.65**^b^	**3.402**^a^	**37.056**^b^	**7.970**^b^	2.517	**19.78**^b^
Stress temperature	0.04	0.978	0.233	0.663	0.334	**3.654**^a^
Organ ^a^ Growing season	**44.643**^b^	**13.945**^b^	**143.504**^b^	**13.936**^b^	**31.730**^b^	1.126
Organ ^a^ Stress temperature	0.991	1.595	0.556	**3.179**^a^	0.266	0.546
Growing season a Stress temperature	1.64	1.648	0.651	0.282	0.275	**5.579**^b^
Organ ^a^ Growing season a Stress temperature	**6.827**^b^	**5.155**^b^	**3.261**^b^	**2.467**^a^	**3.127**^b^	**2.397**^a^

Note: ^a^, ^b^ represent the significance difference at the 0.05 and 0.01 level, respectively; SS: Soluble sugar content

## Discussion

The MDA content is a reflection of lipid peroxidation and is usually used to measure stress-induced damage at the cellular level [27]. In this study, *Kobresia pygmaea* in different months showed responses to temperature changes. It suffered more damage in June with the decreasing incubation temperature than other months. The leaves and roots of *Kobresia pygmaea* in July and August are more able to adapt to temperature changes, and the content of MDA in leaves and roots were lower. Similar in September, decreasing MDA contents in leaves and roots with the decreasing incubation temperature. These results are similar to a previous report [28], indicating that with the decrease of stress temperature, the damage to plant cell membrane is intensified. Meanwhile, the growth stage can also affect the plant adaptivity to temperature changes. The self-repair ability of plant is higher during the July and August, and the MDA content shows a different degree of decline. Our results showed that the MDA content in the roots fluctuated considerably than that in leaves, indicating that the stress defense mechanisms in the roots started earlier and can effectively alleviate the damage to the cell membrane [29].

Osmotic adjustment is an important physiological mechanism for plants to resist low temperature adversity [30]. Soluble sugars plays an important role in the growth cycle of plants [31]. In this study, the soluble sugar content in the leaves and roots of *Kobresia pygmaea* in different months showed an increasing trend with the decreasing incubation temperature. It has been demonstrated that the protection of soluble sugars on plants is affected by stress temperature and time [32]. The increase of proline in plants is beneficial to improve the cold resistance of plants. Changes in proline content of plants are both affected by low temperature tolerance and the characteristics of germplasm resources [33]. In our study, with the decrease of the stress temperature, the proline content in the leaves and roots of *Kobresia pygmaea* in different months changed at different temperatures, indicating that the low temperature response in leaves and roots of *Kobresia pygmaea* was affected by temperature and, organs, and growing season, resulting in no consistency in the changes of proline content in plants [34]. It has been also suggested that the proline content of the three Cattleya varieties showed no significant differences among different stress temperatures [19]. Therefore, the changes in proline content in plants may be affected by multiple factors [35, 36].

SOD, POD and CAT as the enzymatic detoxification system of active oxygen can effectively remove the active oxygen free radicals in the plants [37]. Species, even varieties of the same species may have different cold resistance capabilities [38, 39]. In our study, the SOD activity in different organs of *Kobresia pygmaea* showed different changing trends with temperature among different months. The cold response of plants is a complex process, which can be regulated by a variety of enzymes and non-enzymatic systems [40]. In the process of scavenging active oxygen, SOD often cooperates with POD or CAT [41, 42]. In this study, the activity of SOD in leaves of *Kobresia pygmaea* in June under different temperature stresses were higher than that in other months, and the activities of POD and CAT were high and fluctuated greatly. This pattern may be related to the different changes in physiological indicators caused by the different mechanisms of different organs in response to low temperature [43]. Several studies suggested that the changing trends of protective enzyme activities in plants under low temperature stress are related to the genetic characteristics of the species, the living environment, the intensity of low temperature stress, tissues and organs and other factors [44].

The value of the membership function reflects the stress resistance of the plant. Using this index, previous reports suggested that the lowest tolerability in alfalfa appears at 0 °C [45]. Our results showed that the stress resistance of different organs of plants in different growing seasons were different. Higher temperatures damaged the leaves of plants, while lower temperatures damaged the roots of plants, indicating that different organs of plants have different tolerance to temperature, which may also be a temperature adaptation strategy of plateau plants. The growing seasons have a significant impact on the osmotic adjustment substances such as Pro and MAD and the activities of enzymes such as SOD and POD in plants. Our study also found that the interaction of organs, organs and months, as well as the interactions of organs, months, and stress temperature, have significant effects on physiological indicators. It has been shown that the stress resistance of plants was not only a complex quantitative trait affected by many factors, but also can be affected by growing seasons [46].

## Conclusions

The growth temperature of plants in the Qinghai-Tibet Plateau is relatively low. In our study, we used laboratory experiments to examine the physiological response of *Kobresia pygmaea* to different temperatures. From high temperature(23 °C) to low temperature(0 °C), the osmotic adjustment substances in the leaves and roots of *Kobresia pygmaea* changed greatly in the June and September. SOD and POD activity in the leaves and roots of *Kobresia pygmaea* in different growing seasons fluctuated strongly, while CAT activities were higher in July and August. Although the physiological indicators in leaves and roots can positively reflect high temperature and low temperature, our results showed that higher temperatures were more harmful to plant leaves, and lower temperatures were more harmful to plant roots. Overall, the physiological indices of *Kobresia pygmaea* showed considerable variations to different temperatures. Due to the large temperature differences in our experiments, our findings suggested that the *Kobresia pygmaea* is a well adaptive specie to alpine environment, and the current climate warming may not lead significant changes in the growth of *Kobresia pygmaea*.

## Methods

### Study Area

**Fig. 7:**
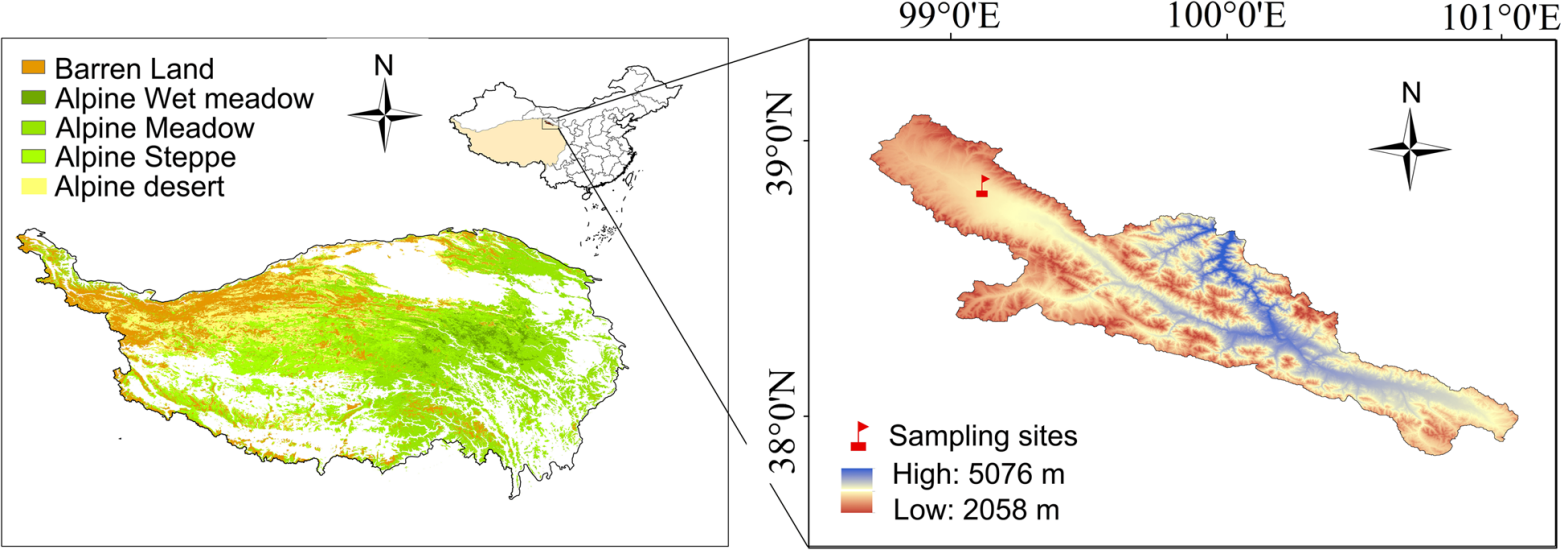
Land cover types and sampling area in the permafrost region of the Qinghai-Tibet Plateau

The study area, the Qilian Mountains are located between 98°75′~99°78′ E, 38°31′~38°82′ N (Fig. 7). The high variation in temperature and uneven distribution of monthly precipitation are the major features of the climate in this region [47]. Climatic data of the a local meteorological station showed that extreme high temperature ranges from 28.5 to 32.4℃ and extreme low temperature varies from −27.8 to −29.0℃. On average, the annual precipitation ranges from 401.9 to 632.3 mm, the annual evaporation varies between 1041.2 mm and 1234.2 mm, and the relative humidity is about 58% [48]. In our study area, the plant community diversity was investigated by sampling method. It was found that *Kobresia pygmaea* was the dominant specie, and others herbaceous plants were *Carex atrata*, *Stipa capillata Linn*, *Elymus nutans Griseb* and *Oxytropis ochrocephala Bunge*. These plants were common species on the QTP and were identified by Lisha Li. The sampling site ( 99°1′ E, 38°48′ N, 3700 m above sea level ) belongs to a *Kobresia pygmaea* meadow, and the coverage of *Kobresia pygmaea* during growth period on site is about 90% ( Fig. 1).

### Experimental design and sample collection

 Our study complied with relevant institutional, national, and international guidelines and legislation, and no specific permits were required to collect the plant samples. The samples were collected monthly from June to September in 2020. According to the five-point sampling method, the 8 pieces of whole plant and soil was excavated about 10 cm×10 cm×10 cm in size with a small shovel at 3700 m above sea level. The samples were stored in 15 cm×10 cm×15 cm flowerpots and brought back to the laboratory for cultivation. The flowerpots were placed in a light incubator, and pre-adapted for 3 days at a temperature of 25 °C, light intensity of level 3 (240 µmol m^−2^ s^−1^), and light time of 16 h/8 h (day/night) [49]. After pretreatment, the samples were placed in a light incubator at 23 °C, and 2 g of *Kobresia pygmaea* leaves and roots were collected at 0 h, 1 h, 3 h, 6 h, 12 h, 24 and 72 h. The samples were rinsed using deionization, dried using absorbent paper, wrapped in aluminum foil, and then stored in liquid nitrogen for laboratory analysis. After the sample collection, the light incubator was cooled at a rate of 2.5° C/h to 10 °C. The samples were also collected at these time intervals. Similarly, the leaves and roots samples of *Kobresia pygmaea* were collected under 4℃ and 0℃.

### Physiological characteristics measurement

The content of malondialdehyde is determined using the thiobarbituric acid (TBA) under acidic and high-temperature conditions, the maximum absorption peak appears at 532nm [50].

Proline content was determined spectrophotometrically using the ninhydrin method. The sample (0.5 g) was placed in a mortar, 5 ml of 3% sulfosalicylic acid was added and then the sample was grinded, homogenized and then transferred to a centrifuge tube, extracted in a boiling water bath for 10 min and cooled to room temperature. After centrifugation at 3000 r·min^−1^ for 10 min, the supernatant (2 ml) was mixed with 4 ml of acid ninhydrin reagent, 2 ml of glacial acetic acid and 2 ml of 3% sulfosalicylic acid, and the reaction mixture was placed in a boiling water bath for 1 h. After cooling to room temperature, 4 ml of toluene was added. After sufficient shaking, the red toluene was placed in a cuvette, and the absorbance was measured at 520 nm. The proline content of the sample was calculated according to the standard curve [51].

The soluble sugar content was determined by the anthrone method. The sample (0.1 g) was put into a 10 ml graduated centrifuge tube, added 4 ml of 80% ethanol, and boiled in water at 80℃ for 30 min with constant shaking. After a centrifugation at 3000 r·min^−1^ for 10 min, the supernatant was collected and placed in a 10 ml test tube. The residue was repeated the above process, and the supernatant was collected. By mixing 1 ml of supernatant and 5 ml of anthrone reagent, the mixture was water bathed in boiling water for 10 min. After cooling, the sample was performed colorimetry at 625 nm. The soluble sugar content was calculated according to the standard curve [52].

Superoxide dismutase (SOD, EC 1.15.1.1) activity was measured by the nitroblue tetrazolium (NBT) method. The sample (0.1 g) was placed in a pre-cooled mortar, added 2 ml of extraction medium to grind, diluted to 10 ml, and centrifuged at 12,000 r·min^−1^ for 30 min. Combined 0.1 ml crude extract, 1.5 ml 50 mM PBS (pH 7.8), 0.3 ml 130 mM L-methionine, 0.3 ml 750 µM NBT, 0.3 ml 100 µM EDTA-Na_2_, 0.3 ml 20 µM riboflavin and 0.5 ml distilled water. The colorimetric tube was placed under a 4000 lx (light intensity) fluorescent lamp for 20 min, and the control tube was placed in a dark place, then the absorbance of each tube was measured at 560 nm. The SOD activity was defined as the amount of enzyme that inhibited the rate of photoreduction of NBT by 50% and was expressed as U mg^−1^ protein [53].

The POD (EC 1.11.1.7) was determined by the guaiacol colorimetric method. The 3 ml reaction mixture (containing 50 ml 0.1 mol·L^−1^ PBS (pH 6.0), 19 µl of 30% H_2_O_2_ and 28 µg guaiacol) and 1ml crude enzyme solution was added to the colorimetric tube. The absorbance change of the reaction solution at 470 nm was measured every minute for a total of 4 min. The POD activity was defined as the amount of enzyme needed to decompose 1 mol of H_2_O_2_ per min at 25˚C [54].

The activity of catalase (CAT, EC 1.11.1.6) was measured by UV absorption method. The sample (0.1 g) was mixed with 25 ml 0.2 mol·L^−1^ PBS (pH 7.8), and centrifuged at 4000 r·min^−1^ for 15 min to obtain a crude enzyme solution. 0.2 ml crude enzyme solution, 1.5 ml PBS (pH 7.8) and 1ml distilled water was mixed and preheated at 25℃, then 0.3ml 0.1 mol·L^−1^ H_2_O _2_ was added, counted immediately after adding 1 tube, and quickly poured into quartz colorimetry tube, measured the absorbance at 240 nm. The CAT activity is defined as a decrease in absorbance of 0.1 per minute [55].

### Statistical analysis

All the assays described above were repeated at least four times on five biological replicates. The membership function method was used to evaluate the adaptability of *Kobresia pygmaea* in different growing seasons to different temperatures. The indices of *Kobresia pygmaea* in response to low temperature stress were further analyzed by the method of multi-factor analysis of variance. We used SPSS 25, Statistic 10 for windows to complete the statistical analysis of the data, and used the Origin9.0 software for data mapping.

Membership function calculation formula: R(X_ij_) = (X_ij_ -X_min_)/(X_max_ -X_min_). R(X_ij_) is the subordinate function value of i variety j index; X_ij_ is the resistance coefficient of a certain breed; X_max_ and X_min_ are the maximum and minimum values of the test varieties in the j index, respectively; The higher the membership function value R, the stronger the plant resistance.

## Supplementary Information

**Additional file 1.**

## Data Availability

All data generated or analyzed during this study are included in this published article and its supplementary information file.

## Abbreviations

QTP: Qinghai-Tibetan Plateau
SS: Soluble sugar
MDA: malondialdehyde
SOD: Superoxide dismutase
POD: peroxidase
CAT: catalase
