# Correction to: Physiological response of *Kobresia pygmaea* to temperature changes on the Qinghai-Tibet Plateau

**DOI:** 10.1186/s12870-022-03452-9

**Published:** 2022-02-10

**Authors:** Haiyan Xu, Lisha Li, Nan Mao, Zipeng Gan, Shouye Xue, Xiaoming Li, Bo Zhang, Guimin Liu, Xiaodong Wu

**Affiliations:** 1 School of Environmental and Municipal Engineering, Lanzhou Jiaotong University, Lanzhou, China; 2 Cryosphere Research Station on the Qinghai-Tibet Plateau, State Key Laboratory of Cryospheric Science, Northwest Institute of Eco-Environment and Resources, Chinese Academy of Sciences, Lanzhou, China

**Correction to: BMC Plant Biol 22, 51 (2022)**

**https://doi.org/10.1186/s12870-022-03428-9**

Following publication of the original article [1], it was noted that due to a typesetting error, authors, Haiyan Xu and Lisha Li were not tagged as equal contributors.

^†^Haiyan Xu and Lisha Li contributed equally.

The original article [1] has been corrected.
